# Supplementary material for: Flexible interlocked porous frameworks allow quantitative photoisomerization in a crystalline solid
Source: Nat Commun. 2017 Jul 24;8:100. doi: 10.1038/s41467-017-00122-5 (PMC5524650; doi:10.1038/s41467-017-00122-5)
Supplement: Supplementary file 1 — Supplementary Information [file 41467_2017_122_MOESM1_ESM.pdf]

File Name: Supplementary Information
Description: Supplementary Figures and Supplementary Table

File Name: Peer Review File
Description:

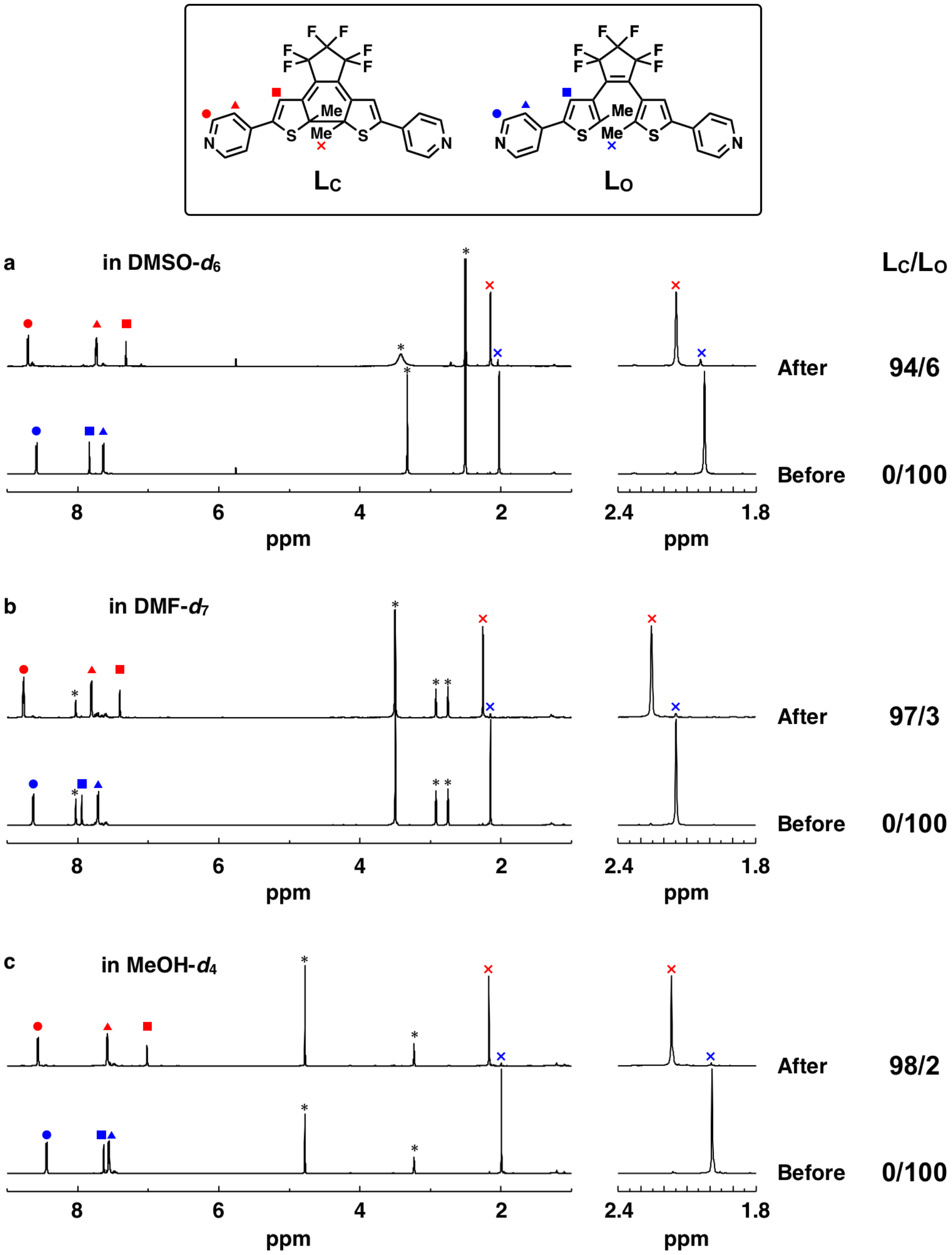

Supplementary Figure 1. Photochemical conversions from L_O to L_C in deuterated solvents. ^{1}H NMR spectra (298 K) of the photochromic ligands before (bottom) and after (top) UV light irradiation in (**a**) DMSO-d_6, (**b**) DMF-d_7, and (**c**) MeOH-d_4. Based on the signals for methyl protons (marked by crosses), the ratios of $\mathbf{L_C}$ to $\mathbf{L_O}$ are estimated to be 94/6, 97/3, and 98/2 in DMSO-d_6, DMF-d_7, and MeOH-d_4, respectively, after 1 h of UV irradiation. The asterisks are the signals of the solvents and water.

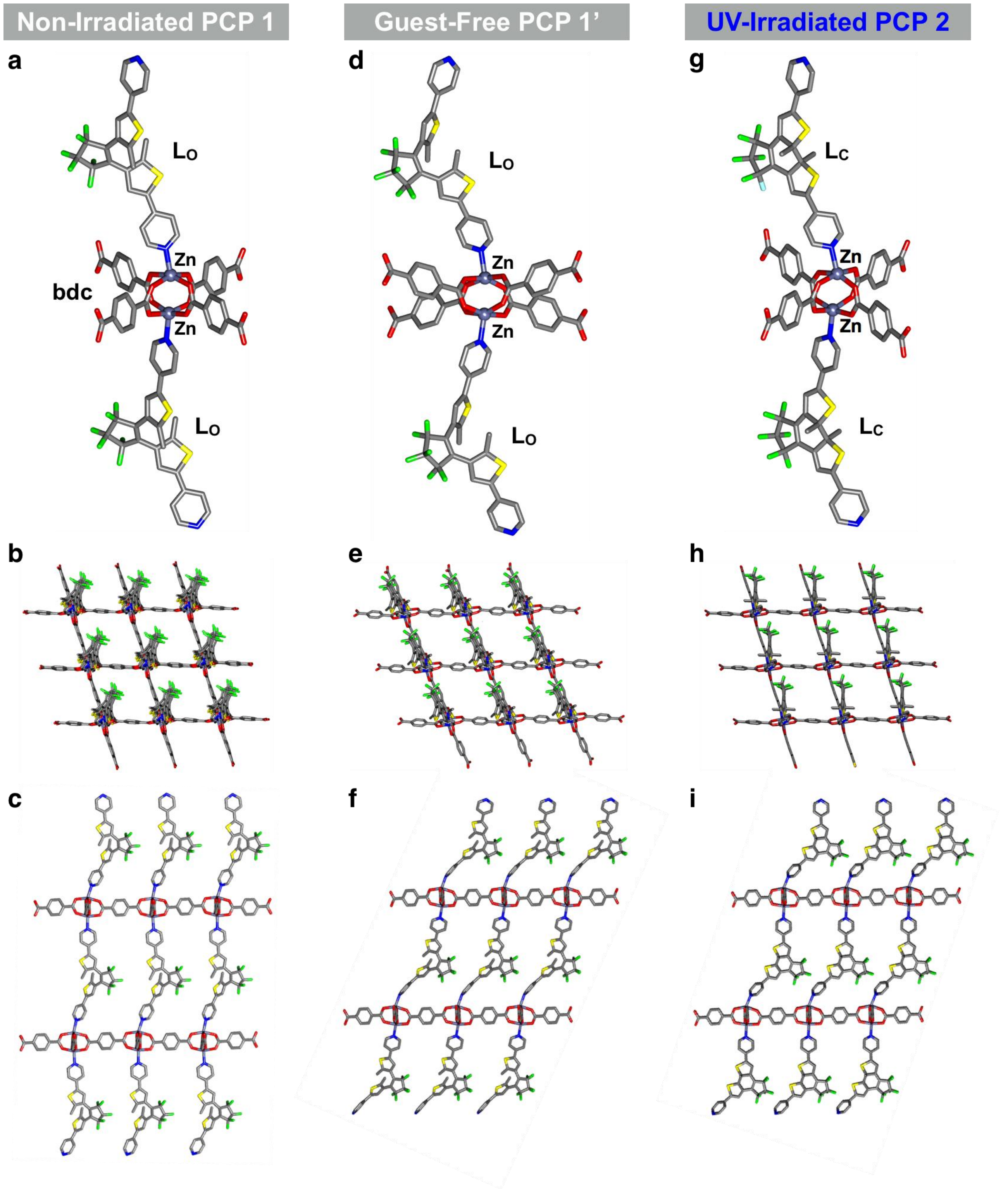

Supplementary Figure 2. Crystal structure of as-synthesized PCP 1 (a-c), guest-free PCP 1’ (d-f), and UV-irradiated PCP 2 (g-i). Atoms are coloured as follows: Zn, cyan; C, grey; N, blue; O, red; S, yellow; F, green. Hydrogen atoms, DMF and water molecules are omitted for clarity. **a**, **d**, **g**, Coordination environment around Zn ions. **b**, **e**, **h**, Top views of pillared-layer structures. **c**, **f**, **i**, Side views of pillared-layer structures.

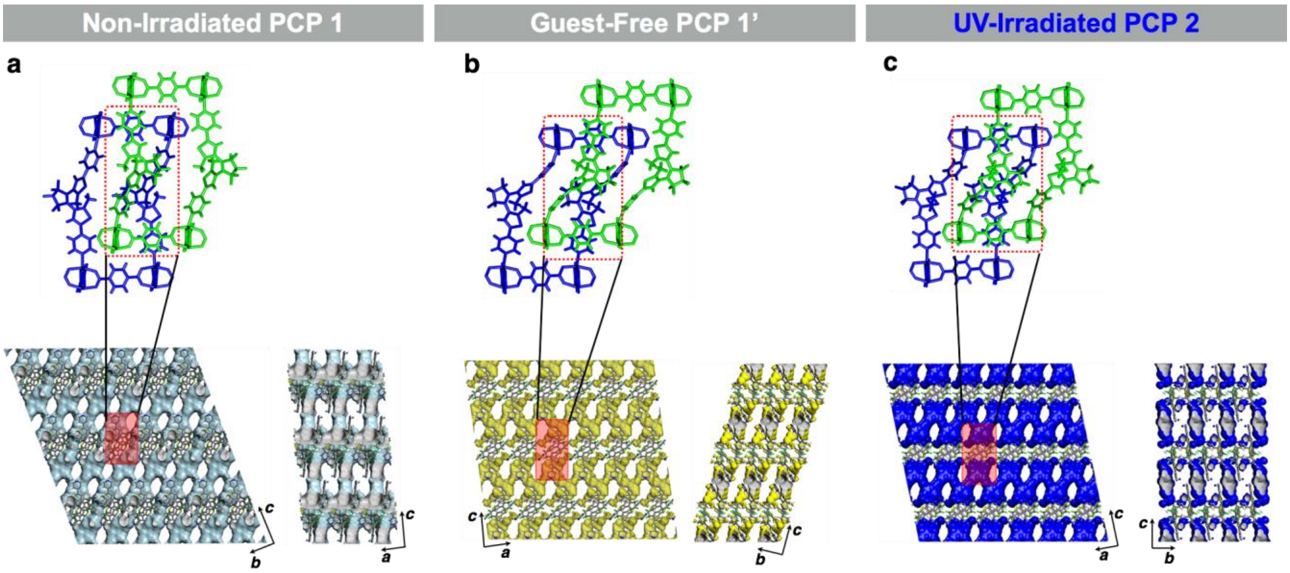

Supplementary Figure 3. Channel structures of as-synthesized 1 (a), guest-free 1' (b), and UV-irradiated 2 (c). The Connolly surfaces (Connolly radius: 1.6 Å) are depicted in pale blue, yellow, and blue in PCPs **1**, **1'**, and **2**, respectively. One of the two-fold interpenetrated frameworks is highlighted in green and the other in blue. In the figures of channels, the layers composed of Zn ions, bdc^{2-}, and guest molecules (DMF and water) are omitted for clarity. The channels in PCP **1** are three-dimensionally connected, while the channels in **1'** and **2** are found in one-dimensional zig-zag shapes.

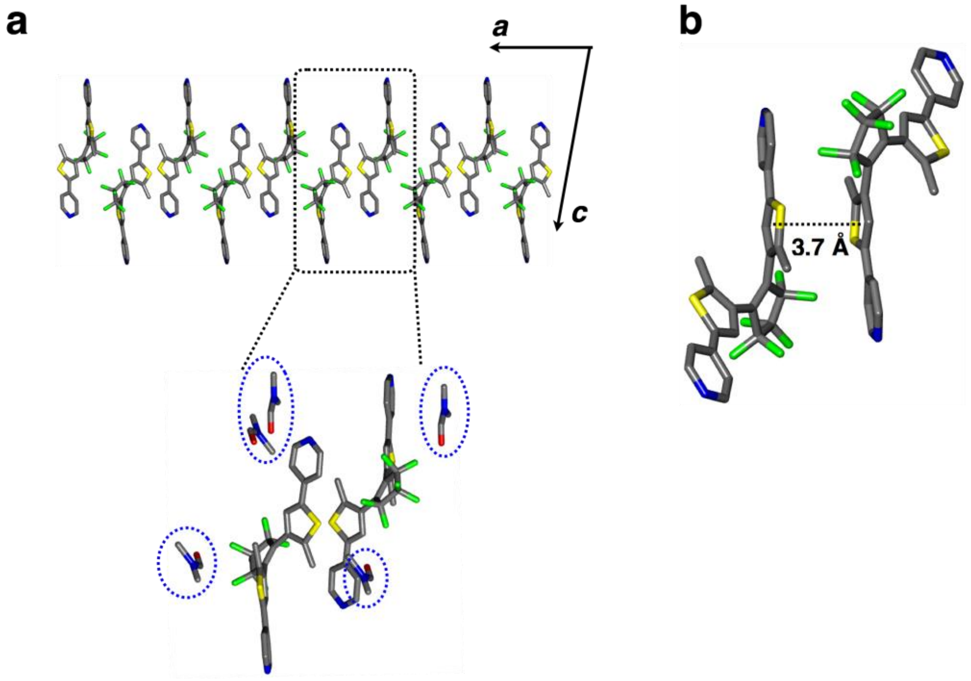

Supplementary Figure 4. The arrangement of Lo in PCP 1. C, grey; N, blue; O, red; S, yellow; F, green. Hydrogen atoms are omitted for clarity. **a**, One-dimensional array of **Lo** along the *a*-axis. **Lo** molecules are surrounded by DMF molecules marked by blue circles. **b**, π - π interaction was found in a pair of neighbouring **Lo** molecules.

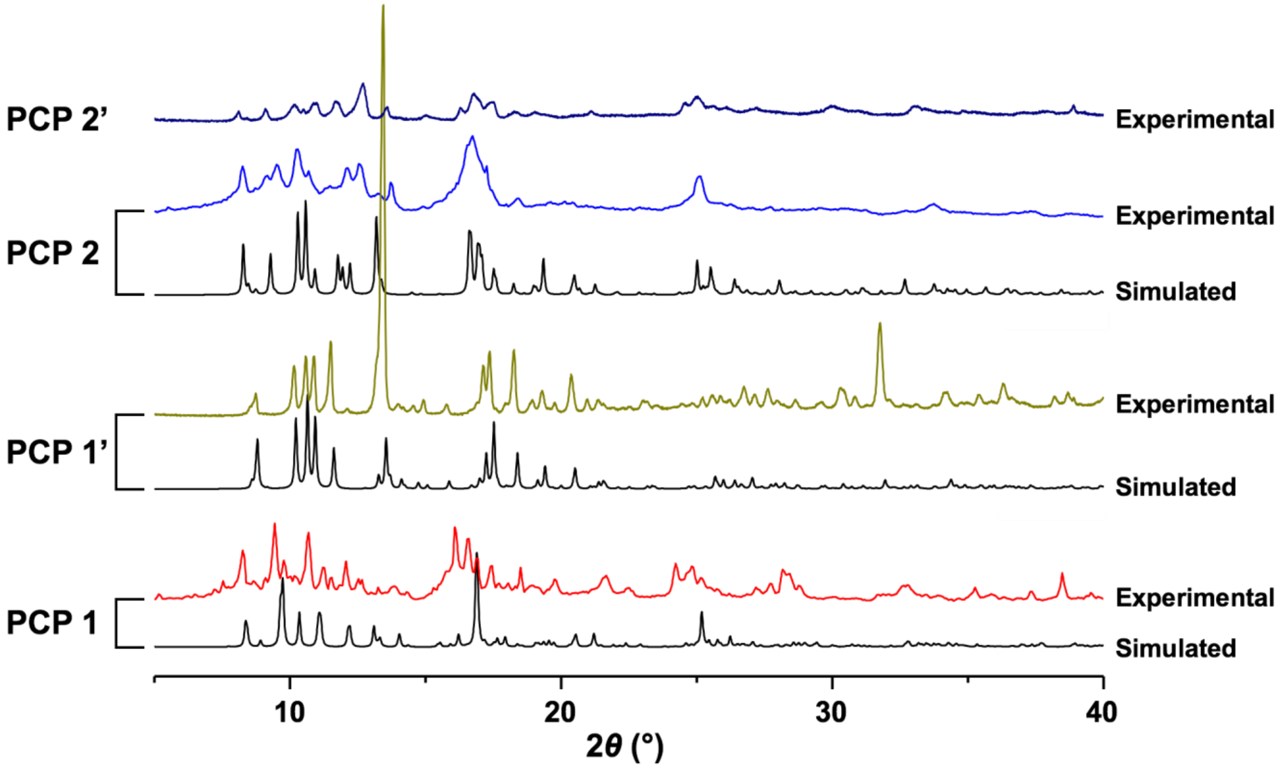

Supplementary Figure 5. XRPD patterns for PCPs as-synthesized **1**, guest-free **1'**, UV-irradiated **2**, and UV-irradiated guest-free **2'**. Data were obtained using Cu $K\alpha$ radiation (λ = 1.54 Å).

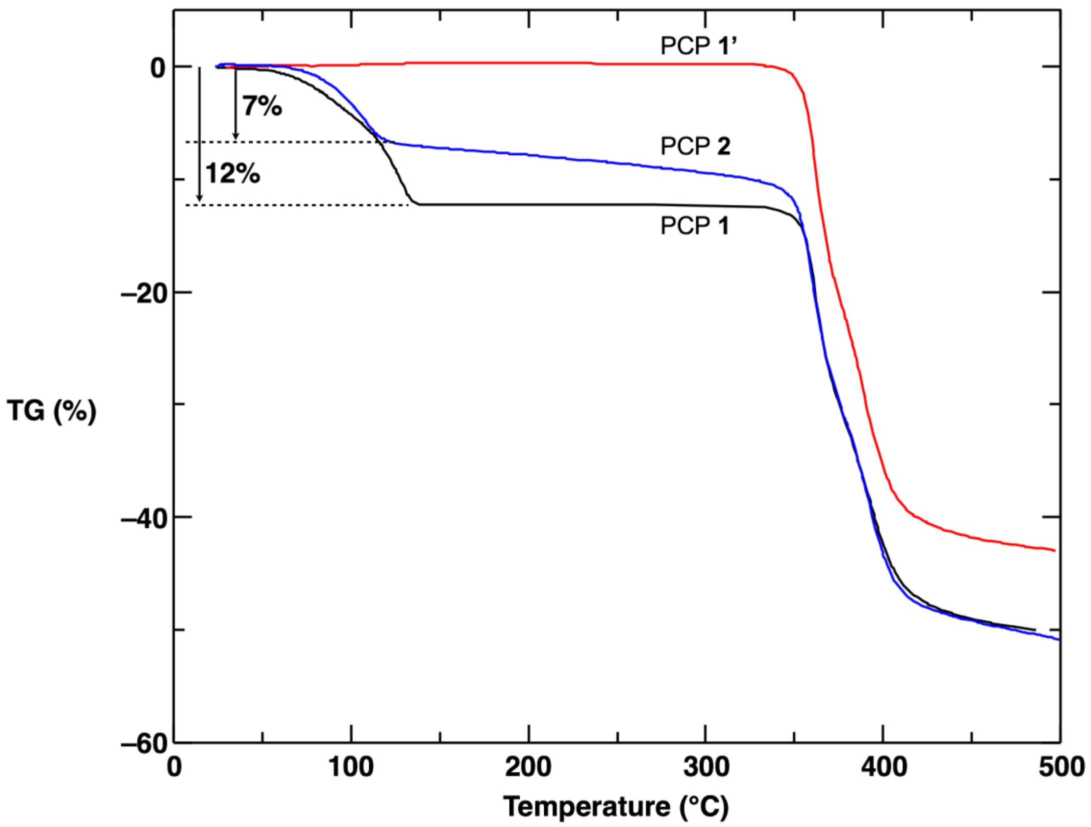

Supplementary Figure 6. TG profiles of PCP 1 (black), 1' (red), and 2 (blue). Weight loss was due to the loss of the guest molecules (DMF and water). Calcd. for PCP **1**: 13.7%, PCP **2**: 6.9%.

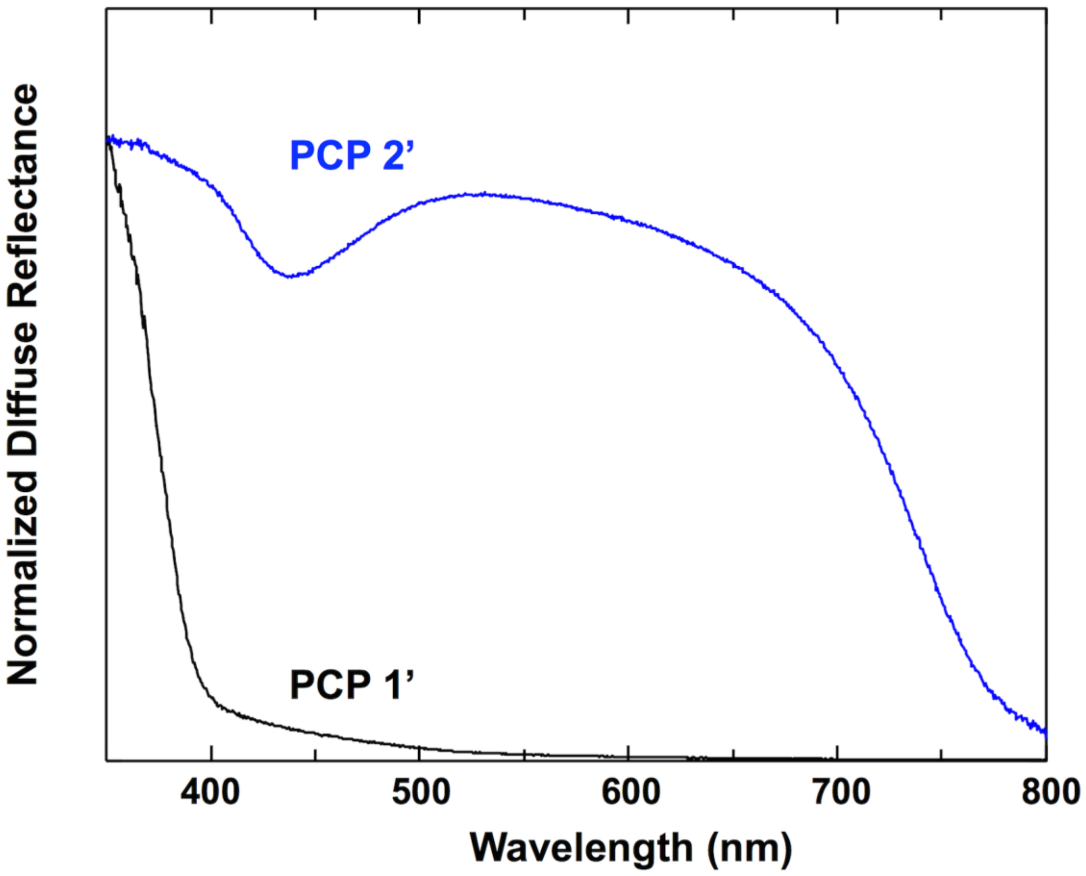

Supplementary Figure 7. Diffuse reflectance spectra of PCP **1'** (black) and **2'** (blue).

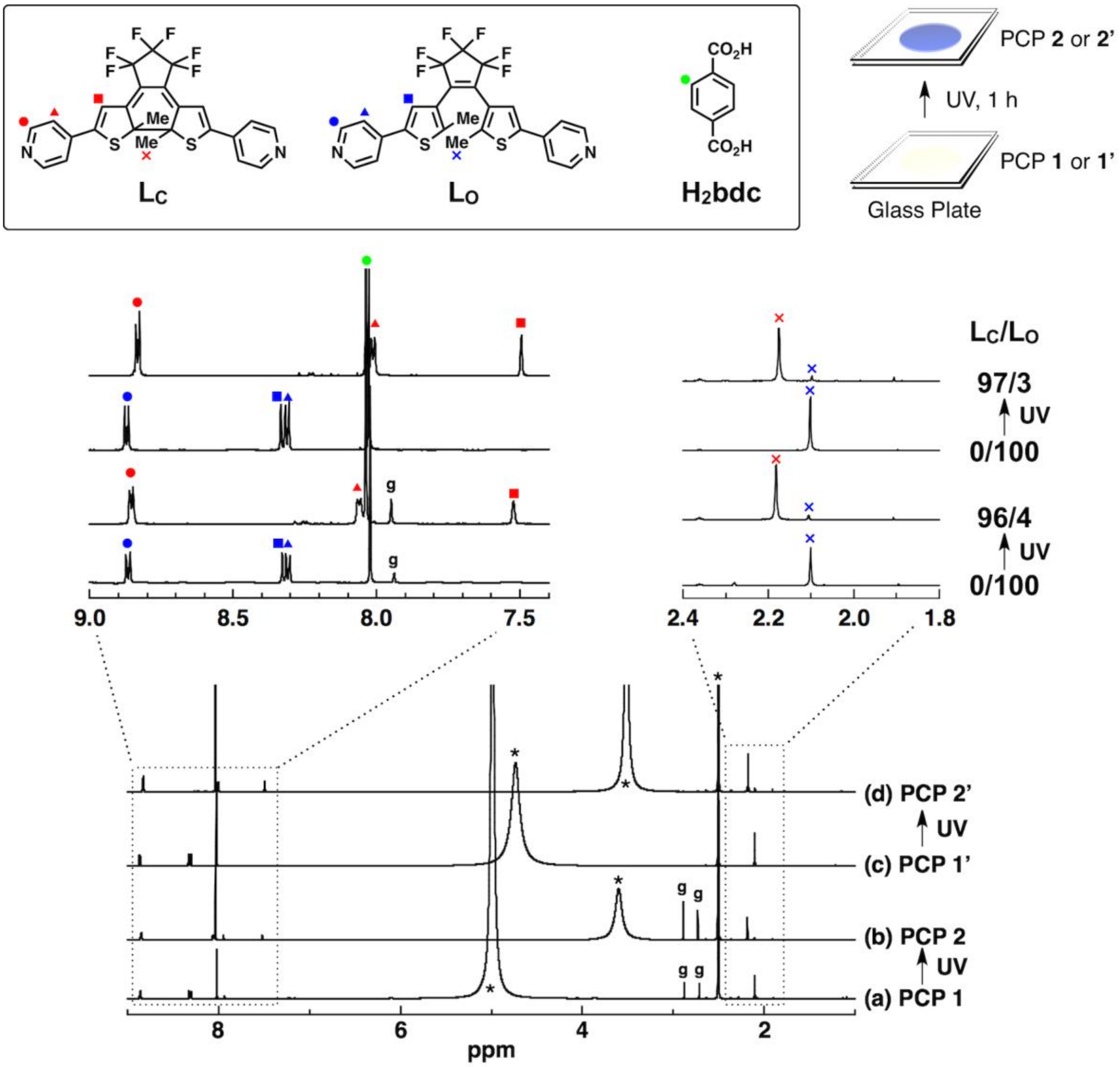

Supplementary Figure 8. Photochemical conversions from LO to LC in the PCP crystals. ^{1}H NMR spectra of (**a**) as-synthesized **1**, (**b**) UV-irradiated **2**, (**c**) guest-free **1'** and (**d**) UV-irradiated guest-free **2'** digested in DMSO-d_6/aq. HCl. The ratios of **LC/LO** in PCP **2** and **2'** are estimated to be 96/4 and 97/3, respectively. The signals marked with an asterisk are assignable to those of the solvents and water. The signals labelled with "**g**" are the protons of the DMF molecules included as a guest. A schematic illustration for the preparation of the samples for ^{1}H NMR spectroscopy is shown on the top.

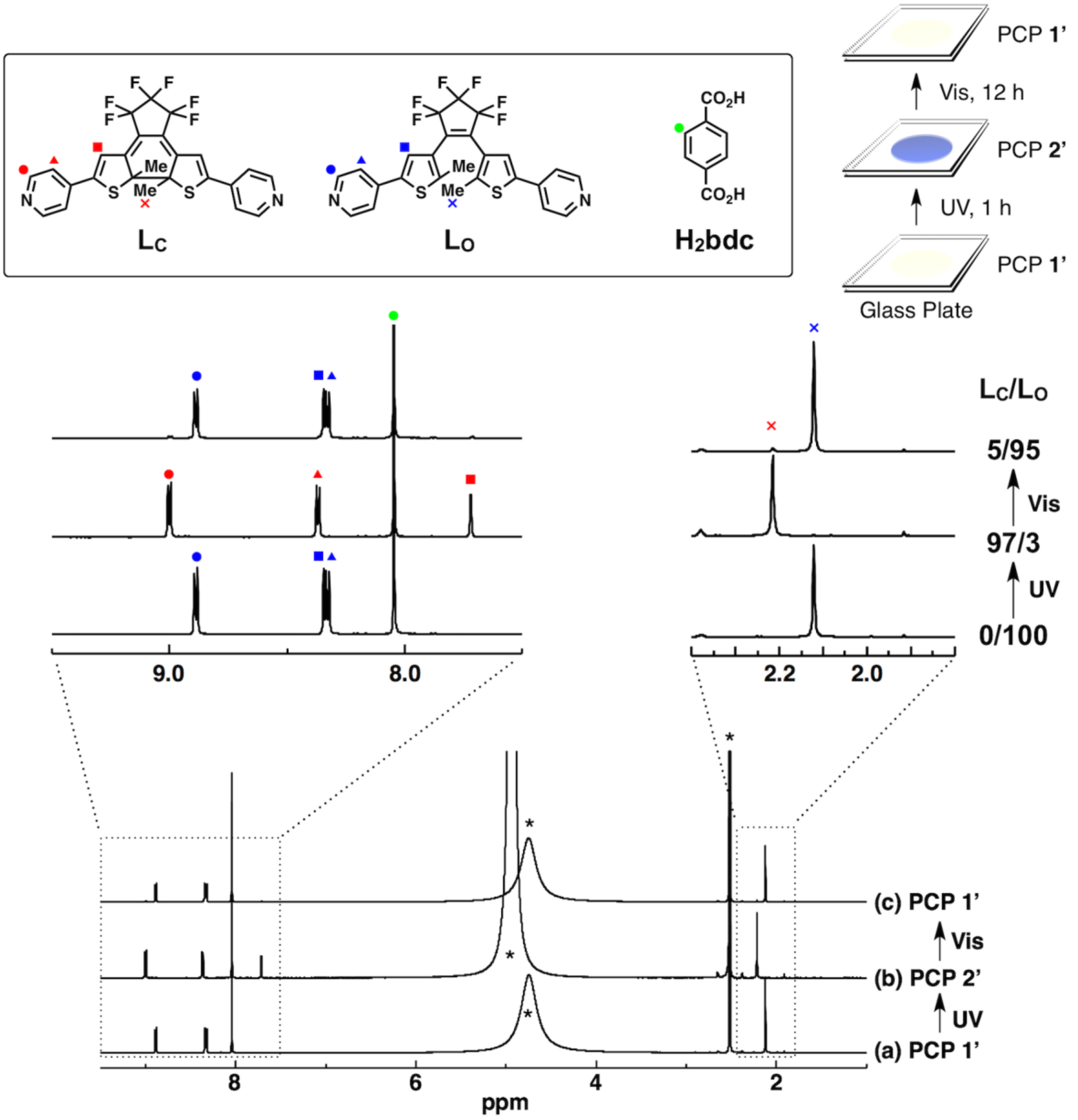

Supplementary Figure 9. Reversible photochemical conversions between L_O and L_C in the PCP crystals. ^{1}H NMR spectra of guest-free **1'** (a) before and (b) after irradiation with UV (PCP **2'**), and (c) after irradiation with UV and visible light (recovered PCP **1'**). The solid substanecs were digested in DMSO-d_6/aq. HCl for ^{1}H NMR spectroscopy. The ratios of L_C/L_O in PCP **2'** and recovered PCP **1'** are estimated to be 97/3 and 5/95, respectively. The signals marked with an asterisk are assignable to those of the solvents and water. A schematic illustration for the preparation of the samples for ^{1}H NMR spectroscopy is shown on the top.

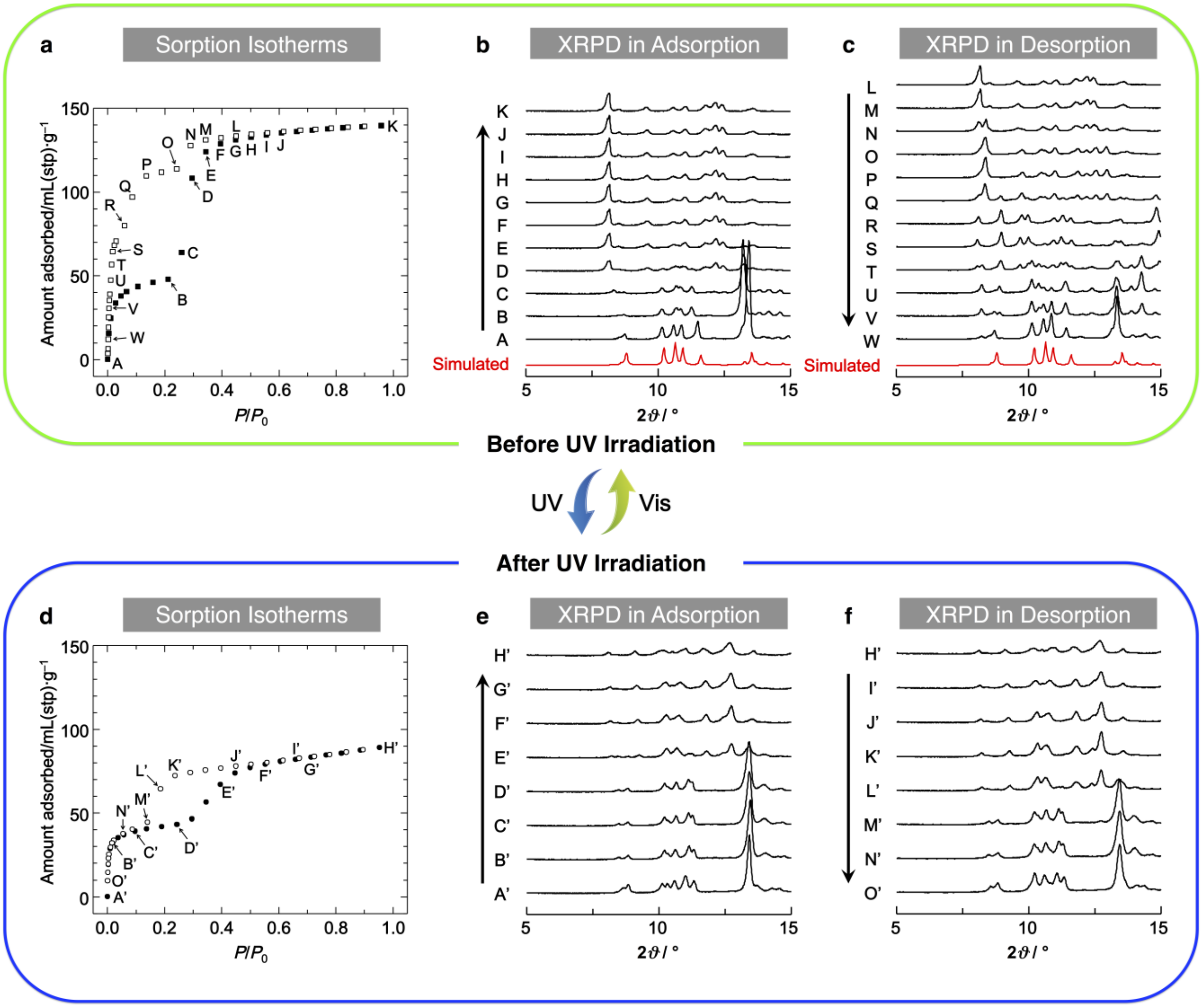

Supplementary Figure 10. Coincident XRPD and CO_2 sorption measurements of PCPs 1' (a-c) and 2' (d-f) at 195 K. a, CO_2 adsorption (filled) and desorption (open) of non-irradiated PCP **1'**. **b**, XRPD patterns measured for the adsorption branch in **a**. **c**, XRPD patterns measured for the desorption branch in **a**. **d**, CO_2 adsorption (filled) and desorption (open) of UV-irradiated PCP **2'**. **e**, XRPD patterns measured for the adsorption branch in **d**. **f**, XRPD patterns measured for the desorption branch in **d**. STP means standard temperature and pressure.

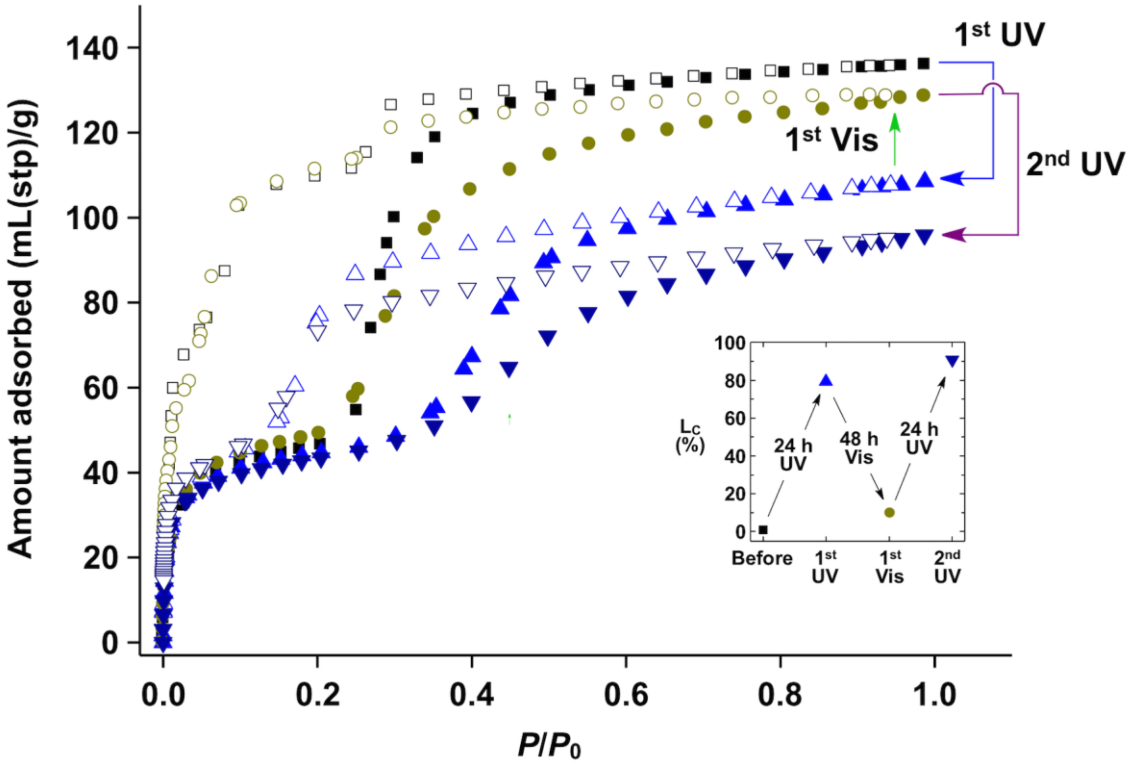

Supplementary Figure 11. Photochemically reversible control of the CO_2 sorption behaviour on PCP 1'. The CO_2 amounts adsorbed on the PCP at $P/P_0 = 0.95$ were found to be 136 (■, initial PCP **1'**), 108 (▲, PCP **1'** after 1st UV irradiation), 129 (●, PCP **1'** after 1st UV and 1st vis light irradiation), and 96 ml(stp)·g^{-1} (▼, PCP **1'** after 1st UV, 1st vis, and 2nd UV irradiation). By taking advantage of the thermal stability of **L**C, we successfully obtained the profile of **L**C contents by digesting the photoirradiated samples with DCl in DMSO-d_6. The inset shows **L**C contents in the solid substances used for the CO_2 sorption experiments. The result indicates that the photomodulation of CO_2 sorption is reversibly achieved by irradiation with UV and visible light.

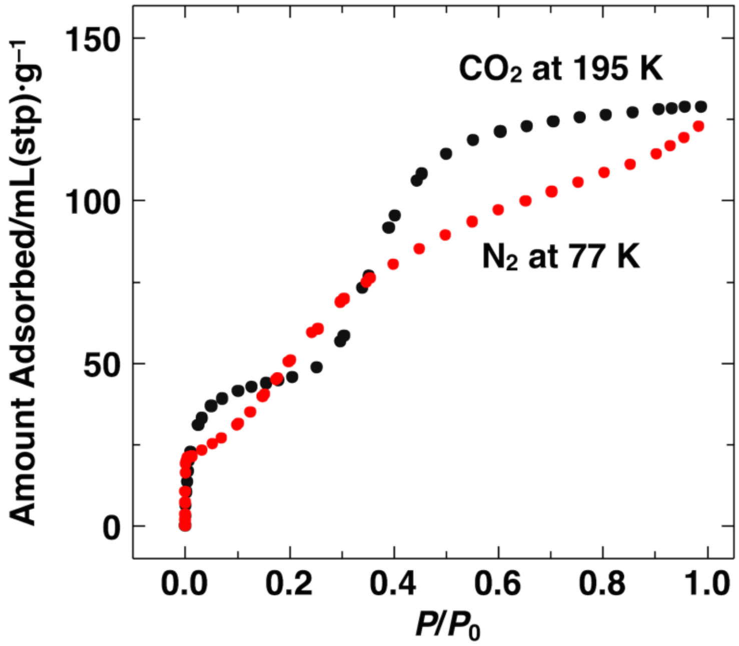

Supplementary Figure 12. Gas sorption isotherms of PCP 1’ for CO_2 (195 K) and N_2 (77 K).

Supplementary Table 1. X-ray crystallographic data for PCP **1**, **1’**, and **2**.

Parameters	PCP **1**	PCP **1’**	PCP **2**
Crystal system	Triclinic	Triclinic	Triclinic
Space group	P−1	P−1	P−1
a, Å	10.920(4)	10.832(3)	10.983(10)
b, Å	21.693(6)	10.893(2)	11.014(12)
c, Å	23.699(6)	20.388(5)	20.55(2)
α, deg	67.91(2)	104.162(18)	88.39(4)
β, deg	79.76(2)	92.972(19)	78.45(4)
γ, deg	76.563(18)	106.805(17)	75.71(5)
V, Å^3	5034(3)	2213.3(9)	2360(4)
Accessible volume, Å^3	1528.4 (30.4 %)	324.5 (14.7 %)	498.4 (21.1 %)
Z	2	2	2
T, K	103	103	100
D_{calcd}, g/cm^3	1.499	1.473	1.484
GOF on F^2	1.266	1.303	1.054
$R_1[I > 2\sigma(I)]$	0.1196	0.1026	0.1533
$R_2[I > 2\sigma(I)]$	0.3223	0.3056	0.4284
Data Completeness	0.959	0.933	0.931
